# Image Exchange: an International Perspective

**DOI:** 10.1007/s10278-021-00540-4

**Published:** 2022-08-24

**Authors:** David S. Mendelson

**Affiliations:** 1 Mount Sinai Health System, New York, USA; 2 Icahn School of Medicine at Mount Sinai, New York, USA; 3 Integrating the Healthcare Enterprise (IHE) - International, Oak Brook, USA

Patient healthcare and related information should move with the patient for the highest quality of care! Such a simple and straightforward concept — everyone agrees and yet the reality around the world is just now beginning to reflect this simple maxim. Today, we expect quick and secure exchange over the internet. Why has this been delayed for imaging and what remains to be done to arrive at a state where this is the norm?

Historical healthcare information is most valuable when the patient presents with a new abnormality. This is certainly true of imaging exams when demonstration of change can ultimately lead to care decisions. Every radiologist learns early on the value of a prior exam when interpreting a new abnormality and offering a differential diagnosis and sometimes a prognostic statement.

I became deeply engaged with these issues as principal investigator of the RSNA ImageShare project [1] sponsored by the National Institute of Biomedical Imaging and Bioengineering (NIBIB) in 2009. At that time, there was great frustration in the overall healthcare community with the CD as the primary mode of image exchange. Why were not we moving images across the Internet, just as we moved photos, video, and music? Technical standards that would have enabled interoperability were not being observed; rather, proprietary solutions were pervasive. NIBIB recognized this issue and made the use of healthcare technical standards a requirement. I and my colleagues dealt with these issues that had stymied the implementation of easy imaging interoperability. We identified the standards that the vendors would need to comply with.

NIBIB also stipulated that the solution we developed needed to demonstrate patient control of their data. Given that focus, we proceeded, along with vendor partners, to build image enabled personal health records (PHRs). However, at that time, the healthcare community was not prepared for easy patient digital access and ownership of their data. We spent several years navigating poorly understood security and HIPAA regulations that governed patient control and access.

The RSNA ImageShare enrolled just over 35,000 patients. The grant terminated in 2015, but I am pleased that some of the participating vendors have continued to offer patients their own accounts in image enabled PHRs. We and the vendor community learned that the technical standards we were promoting worked well, and we gained experience with the security and patient identity issues. As you will read in this issue, these lessons are now finally being applied to image enable health information exchanges (HIEs). HIEs are likely to become the dominant place for image exchange with patient consent.

The above project along with other pilot and demonstration programs showed what could be accomplished. Yet health information interoperability remained stagnant on a national basis. Meaningful use [2] succeeded in building a digital infrastructure across the USA but stopped short of achieving interoperability. The impediments have not been technological in nature but rather sociologic. Motivations for “Information Blocking” include vendors offering proprietary exchange systems hoping to monopolize this space and Integrated Delivery Networks hoping to capture patients and hold them by limiting the outside exposure of the information pertinent to that patient. It has required regulatory actions and the threat of financial penalties to overcome these hurdles. Patient identity, privacy, and consent issues have presented challenges. In the USA, there is no single patient identifier. With the understanding that there are strong philosophical differences of opinion regarding this issue, a single identifier would certainly diminish the issues around confirming patient identity when moving information between disparate organizations. Other parts of the world have government-prescribed single patient identifiers which have made this a non-issue. For those of us without such a unique identifier, patient-matching solutions have evolved but bring about an additional set of technical challenges complicating interoperability solutions and data integrity.

The essential components of interoperability are the same for imaging as for the remainder of health care. These include patient consent, authentication, confirmation of patient identity, standards for transmission, and a viewer. The SOAP-based IHE XDS–based standards are frequently employed. FHIR is emerging as an evolutionary standard and should be integrated into the workflows as we move forward. Technology will always evolve, and the solutions we put in place should have an intelligent path forward to reflect this without major disruption.

Throughout this issue of JDI, we will see that interoperability has achieved different levels of penetration around the world. Varying approaches to achieve the same end are discussed. We will take the reader from the global level down to the work required at the local department to enable the transparent and secure exchange of imaging exams. Though our focus is image exchange, it should be placed in the broader context of healthcare interoperability, and we have included discussions that will provide the reader with this context.

We hope that the reader of this issue will join us with an optimistic view that we are on the verge of a new era where we have raised the standard of care by putting the image and other healthcare information where it is required, at the current point of care.
